# 

**Published:** 1843-09-02

**Authors:** 


CONTENTS.

A Course of Clinical Lectures, delivered at the Hôtel Dieu, Paris, for the Session 1842–'43. By A. F. Chomel, M.D. Lecture 10, - - - - - - 193

Case of presentation of the Anterior Fontanelle. By John A. Elkinton, M.D. 197

*Bibliographical Notices.*

Minor Surgery; or Hints on the Every-day Duties of the Surgeon. By Henry H. Smith, M.D., Lecturer on Minor Surgery, &c. - - - - - - - 198

On the Theory and Practice of Midwifery. By Fleetwood Churchill, M.D., &c. &c. With Notes and Additions. By Robert M. Huston, M.D., Professor of Materia Medica and General Therapeutics in the Jefferson Medical College of Philadelphia, &c. - - - - - - - - 198

The Dispensatory of the United States of America. By George B. Wood, M.D. Professor of Materia Medica and Pharmacy in the University of Pennsylvania, &c. &c., and Franklin Bache, M.D., Professor of Chemistry in Jefferson Medical College of Philadelphia. Fifth edition, enlarged and revised, - 199

*Editorial.*

Researches on the frequency of Cancer, 200

*Retrospect of the Medical Sciences.*

Dr. Ricord on the treatment of Gonorrhœa, 202

Dr. Ricord on External Gonorrhœa, - 202

Pathological Researches into the Local Causes of Deafness, based on one hundred and twenty dissections of the human Ear. By Joseph Toynbee, M.D., &c. 203

Plea of Insanity in criminal cases, - 204

